# Metformin and Cancer, an Ambiguanidous Relationship

**DOI:** 10.3390/ph15050626

**Published:** 2022-05-19

**Authors:** Sarah J. Skuli, Safwan Alomari, Hallie Gaitsch, A’ishah Bakayoko, Nicolas Skuli, Betty M. Tyler

**Affiliations:** 1 Division of Hematology and Oncology, Department of Medicine, Perelman School of Medicine, University of Pennsylvania, Philadelphia, PA 19104, USA; sarah.skuli@pennmedicine.upenn.edu (S.J.S.); nicskuli@pennmedicine.upenn.edu (N.S.); 2 Hunterian Neurosurgical Research Laboratory, Department of Neurosurgery, Johns Hopkins University School of Medicine, Baltimore, MD 21205, USA; salomar1@jhmi.edu (S.A.); hgaitsc1@jhmi.edu (H.G.); 3 NIH-Oxford-Cambridge Scholars Program, Wellcome—MRC Cambridge Stem Cell Institute and Department of Clinical Neurosciences, University of Cambridge, Cambridge CB2 1TN, UK; 4 Stem Cell and Xenograft Core, Perelman School of Medicine, University of Pennsylvania, Philadelphia, PA 19104, USA; aishahb@sas.upenn.edu

**Keywords:** metformin, diabetes, cancer metabolism, AMPK, PI3K, therapeutics, drug repurposing

## Abstract

The deregulation of energetic and cellular metabolism is a signature of cancer cells. Thus, drugs targeting cancer cell metabolism may have promising therapeutic potential. Previous reports demonstrate that the widely used normoglycemic agent, metformin, can decrease the risk of cancer in type 2 diabetics and inhibit cell growth in various cancers, including pancreatic, colon, prostate, ovarian, and breast cancer. While metformin is a known adenosine monophosphate-activated protein kinase (AMPK) agonist and an inhibitor of the electron transport chain complex I, its mechanism of action in cancer cells as well as its effect on cancer metabolism is not clearly established. In this review, we will give an update on the role of metformin as an antitumoral agent and detail relevant evidence on the potential use and mechanisms of action of metformin in cancer. Analyzing antitumoral, signaling, and metabolic impacts of metformin on cancer cells may provide promising new therapeutic strategies in oncology.

## 1. Introduction

The history of the biguanide, metformin (molecular formula C4-H11-N5, Table 1), is linked to *Galega officinalis* and is also known as French lilac or Italian fitch. The *Galega officinalis* represents a traditional herbal medicine that was found to lower blood glucose in 1918 [1]. Guanidine derivatives were used to treat diabetes mellitus (DM) in the 1920s and 1930s but with the availability of insulin were discontinued due to their toxicity [2]. During World War II and throughout the search for antimalarial agents, metformin was re-discovered and determined to lower blood glucose levels [3,4]. The French physician-scientist Jean Sterne was the first to report the use of metformin to treat DM in 1957 and named the compound Glucophage, which means glucose eater [5]. Since its introduction, metformin has become the most prescribed glucose-lowering drug worldwide [2]. 

In 1998, the UK Prospective Diabetes Study (UKPDS), a prospective randomized trial of 5100 type 2 DM patients who received glucose-lowering treatment for more than a decade showed reduced cancer risk [6]. Subsequent large database analyses have reported lower incidence of certain types of cancer among diabetic populations taking metformin despite data indicating that these diabetic populations were overall more prone to developing cancer. This has led to a deeper investigation into the role of metformin in cancer [7,8]. Here, we review five years of updated literature on metformin’s antineoplastic activity, its mechanisms of action, as well as current limitations and future directions for the repurposing of metformin in the treatment of cancer. 

## 2. Metformin in Cancer

To date, there are over 50 recent or active clinical trials investigating the use of metformin in human malignancies (Table 2). Total daily dose of oral metformin in these clinical trials ranges from 500 to 3000 mg. This range reflects the previously established dosing strategy used to treat patients with type 2 DM, with gastrointestinal (GI) toxicity limiting use beyond 2500 mg per day [9]. In future clinical trials, we suggest aiming to achieve the maximum tolerated dose of 2500 mg per day given the majority of preclinical studies required high concentrations of metformin to achieve anti-cancer activity [10]. Furthermore, we recommend planned dose escalation to allow for GI habituation as well as allowance of dose interruptions and reductions for drug toxicity to reflect real-world practices.

### 2.1. Glioma

While there remains a lack of high-level evidence describing the specific role of metformin in patients with brain tumors, available literature has reported several advantages of repurposing metformin to be used in the management of glioma. Systemically administered drugs must be able to cross the blood–brain barrier (BBB) to effectively treat brain tumors. Using a rat model, orally administered metformin was found to penetrate the BBB at a high rate with biodistribution throughout the central nervous system [49]. Furthermore, metformin reduces vasogenic brain edema and the neurological symptoms that accompany brain tumors [50]. There has also been recent effort to characterize the subpopulations of glioma patients that would benefit most from metformin. A recent retrospective study of 1093 patients with high-grade glioma from a population-based clinical cancer registry in Germany reported a survival benefit from metformin in patients with World Health Organization (WHO) grade III glioma [51]. The benefit in WHO grade III glioma is attributed to the high frequency of isocitrate dehydrogenase (IDH) mutations, which can increase the vulnerability of tumor cells to therapeutic interventions targeting glutamine and mitochondrial metabolism [52].

### 2.2. Breast Cancer

Despite promising preclinical studies demonstrating the synergistic effects of metformin and breast cancer chemotherapeutics [53], several clinical trials investigating the addition of metformin to traditional treatment regimens did not result in improved efficacy. Negative results were seen with trials using metformin and aromatase inhibitors in hormone receptor (HR)-positive breast cancer [11], metformin/doxorubicin/cyclophosphamide in human epidermal growth factor receptor 2 (HER2)-negative breast cancer [12], and metformin and erlotinib in patients with metastatic triple negative breast cancer [13]. Another trial of nondiabetic patients receiving several different chemotherapeutic agents for metastatic breast cancer found that the addition of metformin had no effect on progression free survival (PFS) or overall survival (OS) [14]. However, there have been some positive results using metformin to treat breast tumors. Metformin monotherapy has been found to reduce the likelihood of significant tumor enlargement in women with breast fibroadenomas [15]. Interestingly, subanalysis of a trial featuring HER2-positive breast cancer patients revealed that metformin-treated DM participants had better prognoses compared to patients not treated with metformin, whereas the outcomes of patients with HR-negative cancers were not affected by DM status [16]. Furthermore, combined therapy with everolimus, exemestane, and metformin provided moderate clinical benefit in overweight and obese patients with metastatic, HR-positive, HER2-negative breast cancer [17].

### 2.3. Lung Cancer

The use of metformin in non-small cell lung cancer (NSCLC) is the focus of many conflicting clinical trial results. Based on preclinical studies indicating that metformin can sensitize lung cancer cells to tyrosine kinase inhibitors (TKIs), a combination of gefitinib, a TKI-targeting mutant epidermal growth factor receptor (EGFR), and metformin was tested in nondiabetic NSCLC patients. However, co-treatment resulted in non-significantly worse outcomes for NSCLC patients in terms of PFS and OS [18,54]. In contrast, a trial comparing EGFR-TKI combination treatment with metformin versus EGFR-TKI monotherapy in advanced NSCLC found that there was a significant survival benefit to the addition of metformin [19]. It is possible that the synergistic effect of metformin and EGFR-TKIs is only observable in patients with higher body mass index (BMI), thereby resulting in conflicting phase II trial results [55]. These mixed results extend beyond that of EGFR-TKI combination therapies. Two studies examining the impact of combining metformin with chemoradiation found that metformin resulted in either no survival benefit [20] or worse outcomes, potentially due to drug–drug interactions [21]. Others have reported PFS and/or survival benefits in diabetic NSCLC patients treated with metformin in combination with chemotherapy [22,56,57]. A recent meta-analysis concluded that more randomized clinical trials, particularly those incorporating time-dependent analyses in nondiabetic patients, are necessary to determine the association between metformin and OS in NSCLC [58]. 

### 2.4. Colorectal Cancer

Clinical use of metformin to suppress polyp formation and proliferation in the rectal mucosa of nondiabetic, obese patients with a history of colorectal adenoma has been unsuccessful to date [23]. Furthermore, a subanalysis from the large scale Three or Six Colon Adjuvant (TOSCA) trial found that neither metformin use nor DM status were associated with survival outcomes in colorectal patients receiving adjuvant chemotherapy post-resection [24]. Despite these negative findings, a recent study suggests the potential use of metformin alongside irinotecan for disease control in refractory colorectal patients [25].

### 2.5. Esophageal Cancers

Metformin dosing below the anti-cancer threshold may still activate the tumor immune microenvironment in animal models and patients with esophageal squamous cell carcinoma [59], which in turn may be beneficial for priming patients for subsequent immune checkpoint inhibitor treatment.

### 2.6. Kidney Cancer

Retrospective analysis of clinical trials involving metastatic renal cell carcinoma (mRCC) patients found that the addition of metformin to the TKI, sunitinib, in DM patients was associated with an improved OS compared to use of other diabetic agents [60]. Another retrospective study found that, regardless of diabetic status, the addition of metformin to sunitinib or an alternative TKI, pazopanib, in mRCC patients resulted in a PFS and OS benefit [61].

### 2.7. Liver Cancer

A large, retrospective study comparing diabetic patients receiving sulfonylureas *versus* metformin revealed a strong inverse correlation between metformin use and incidence of hepatocellular carcinoma (HCC) (56% risk reduction), indicating the potential use of metformin as a preventative agent for liver cancer. No association was observed for several other solid tumors after adjusting for BMI and level of glycemic control [62]. Metformin treatment may enhance the benefit of certain interventions, as was demonstrated in a retrospective analysis of patients undergoing Yttrium-90 radioembolization segmentectomy for non-resectable HCC [63]. However, metformin use does not appear to affect HCC recurrence in diabetic patients following initial resection [64]. 

### 2.8. Bladder Cancer

A retrospective analysis of diabetic patients with Bacillus Calmette–Guerin (BCG)-treated, non-muscle-invasive bladder cancer (NMIBC) found that metformin use was associated with increased disease-specific survival and OS [65]. Exploiting the fact that metformin accumulates in the urine prior to excretion, an ongoing trial is testing oral metformin treatment in patients with NMIBC [26]. The high upper limit on metformin dosing in this study (3000 mg daily) may allow for observation of tumor effects not seen in studies using lower doses.

### 2.9. Ovarian Cancer

The effect of metformin on epithelial ovarian cancer (EOC) patient outcomes is ambiguous. A clinical trial in China found that addition of metformin to the traditional therapy for EOC had no impact on PFS [27]. However, a US trial in nondiabetic EOC patients found that neoadjuvant metformin treatment resulted in better-than-expected OS as well as a significant reduction in cancer stem cells [28]. A recent dose escalation study demonstrated that the combination of metformin and paclitaxel/carboplatin is well-tolerated [29]. 

### 2.10. Pancreatic Cancer

A meta-analysis of 21 studies found that metformin treatment was associated with a survival benefit in patients with concurrent DM and pancreatic cancer (PC), specifically for patients at early and intermediate PC disease stages [66], suggesting its potential as an adjuvant chemotherapeutic.

### 2.11. Prostate Cancer

Clinical studies in metastatic, castration-resistant prostate cancer patients show that the addition of metformin is not able to rescue resistance to anti-androgen agent, abiraterone [30], nor is it able to improve survival or response outcomes when combined with a chemotherapy agent, docetaxel [31]. A recent trial combining metformin with a different anti-androgen agent, bicalutamide, in overweight and obese prostate cancer patients found that this paired treatment had no effect on PSA levels compared to bicalutamide alone [32]. 

### 2.12. Skin Cancer

In the treatment of metastatic melanoma, neither metformin monotherapy [33] nor combination with immune checkpoint inhibitors (anti-PD-1 and anti-PD-1/anti-CTLA-4) [67] has been shown to improve patient outcomes.

### 2.13. Uterine Cancer

Pre-hysterectomy metformin treatment in women with endometrial cancer (EC) has yielded mixed results; one study found no anti-cancer effects [34], while others suggest that metformin reduces tumor proliferation [35] and promotes anti-tumor effects by altering EC steroid receptor signaling [36]. These pre-surgical study designs are limited due to the short treatment period and small number of patients enrolled. A recent meta-analysis concluded that metformin does not function as an anti-proliferative agent in EC and is not a beneficial adjunct therapy to progesterone therapy for EC patients seeking to spare their fertility [68], though this latter point is still being investigated in an ongoing clinical trial in Japan [37]. 

### 2.14. Acute Myeloid Leukemia

A retrospective hospital cohort study found that though metformin users did not fare better than non-users in OS and disease-free state, they did far better than insulin users. Insulin users were found to have a two-fold increase in the risk of death and an 85% greater risk of relapse [69].

### 2.15. Chronic Myeloid Leukemia

In a single center observation study, metformin use in combination with a TKI was associated with 100% cytogenetic response (CCyR) compared to only 73.6% of single agent TKI [70]. Patients receiving a TKI with or without metformin were able to achieve major molecular response (MMR) as well as complete molecular response (CMR), however, metformin users achieved this within a shorter period of time with a median time to response of 11.1 months and 37.4 months, respectively, compared to 19.5 months and not reached in the control group [70]. Furthermore, CML leukemic stem cells (LSCs) have been shown to have increased mitochondrial oxygen consumption compared to hematopoietic stem cells (HSCs) [71], which could be specifically targeted by metformin [70]. 

### 2.16. Acute Lymphoblastic Leukemia

In a prospective study of 102 patients with de novo Philadelphia-negative B-cell ALL, metformin use was associated with a lower risk of therapeutic failure (odds ratio (OR) 0.07, 95% confidence interval (CI) 0.0037–1.53) and early relapse (OR 0.05, 95% CI 0.0028–1.153) [72]. Furthermore, the patients who benefited most were those with high expression of multi-drug resistant protein, ATP binding cassette subfamily B member 1 (ABCB1) [72]. In a small phase I clinical trial of ALL patients, the addition of metformin to standard chemotherapy was well-tolerated and yielded responses in a heavily pretreated population, with 56% achieving a complete response (CR) [73].

### 2.17. Myelodysplastic Syndrome

In a single prospective study, no mortality benefit was detected among myelodysplastic syndrome patients receiving metformin or sulfonylureas [74]. 

### 2.18. Lymphoma

In a population-based case-control study and two large, retrospective analyses, there were no significant correlations between metformin use and disease progression or survival in patients with non-Hodgkin lymphoma (NHL) [75,76,77]. However, in a Taiwanese study using a database of over 600,000 newly diagnosed DM patients enrolled in the National Health Insurance database, metformin initiators consistently had a lower risk of NHL [78]. Furthermore, in a retrospective case-control study of DM patients with diffuse large B-cell lymphoma (DLBCL) treated with or without metformin, metformin was associated with improved response to immunochemotherapy [79]. The metformin group had CR and objective response rates (ORR) of 84% and 88%, respectively, compared to control groups, which had rates of 48% and 68% [79]. Additionally, a retrospective case–control study found that CR was achieved in 92% of DLBCL patients on metformin, compared to 54% of control subjects [80]. This data was corroborated by a retrospective study of DLBCL patients with diabetes in which metformin use was associated with improved PFS from 60 to 90 months and OS from 71 to 100 compared to diabetic patients not on metformin [81]. 

### 2.19. Multiple Myeloma

High levels of insulin and a history of DM are poor prognostic indicators for patients with multiple myeloma (MM) [82]. However, within the DM population, metformin was associated with a decreased incidence of death from MM [82]. Metformin use has also been associated with decreased progression of monoclonal gammopathy of unknown significance (MGUS) to MM [83,84]. The current risk of progression to MM is 1% per year in MGUS patients [85]. In a retrospective cohort study from the US Veterans Health Administration database that followed patients diagnosed with MGUS for a total of 10 years, 3% of metformin users progressed to MM compared with 5% of non-users [83]. Among those who did progress to MM, the individuals on metformin progressed in an average of 71 months compared to 47 months in non-users. A similar benefit was found in a matched case–control study from a population-representative database of 11,000,000 individuals treated over an 18-year period in the United Kingdom, but only for those who had received metformin for at least two years [83].

## 3. Metformin, Mechanism of Action

### 3.1. Anti-Cancer Activity of Metformin

In multiple malignancies, metformin has been shown to exert anti-cancer properties, such as decreased proliferation, cell cycle arrest, and induction of apoptosis and/or autophagy [86,87,88]. More recently, it has also been established that metformin can induce alternative forms of cell death, such as pyroptosis, which involves an inflammatory, caspase 1-dependent programmed cell death. Metformin has been shown to induce pyroptosis through adenosine monophosphate-activated protein kinase (AMPK)-dependent activation of sirtuin 1, a nicotinamide adenine dinucleotide (NAD+)-dependent deacetylase, and downstream nuclear factor kappa B (NF-kB) expression [89]. In breast cancer cell lines, metformin has also been shown to induce oxidative stress-dependent necroptosis, which was rescued with necroptosis inhibitors [90]. In in vitro and in vivo models of breast cancer, metformin was found to induce ferroptosis, which is a non-apoptotic form of cell death that involves iron-dependent accumulation of lipid oxidation and depletion of plasma membrane polyunsaturated fatty acids [91,92]. Ferroptosis was induced by upregulation of miRNA-324-3p expression and subsequent downregulation of glutathione peroxidase 4, which is a glutathione-dependent antioxidant enzyme that prevents ferroptosis [91]. Finally, metformin can also induce mitophagy in a cervical cancer cell line [93].

In addition to tumor-killing properties, cancer drug development has also focused on decreasing metastatic spread as well as recurrence post-treatment. Metformin has recently been found to decrease cell motility and invasion while increasing cellular adhesion in multiple solid tumor models [94,95,96,97,98,99]. Furthermore, metformin could specifically target cancer stem cells [96,100,101,102,103,104,105,106,107,108,109]. The mechanisms by which cancer stem cells were targeted varied but included targeting of mitochondrial respiration in osteosarcoma stem cells [100], inhibition of stem cell markers, specifically CD133 in HCC and oral cancer cell lines [102,104] and CD47 in breast cancer [105], and regulation of crucial transcription factors [103,110].

### 3.2. Mechanisms of Metformin’s Anti-Cancer Activity

**Figure 1:**
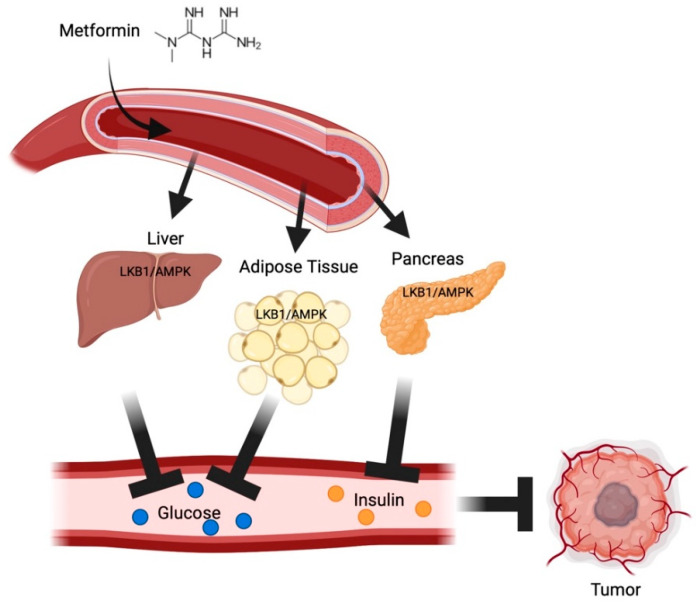
Overview of metformin’s systemic effects on tumor growth. Metformin’s activation of the LKB1/AMPK pathway in hepatocytes and adipocytes, and in the pancreas, leads to reduced blood glucose and insulin availability, respectively. Decreased glucose and insulin availability can slow tumor growth and progression. LKB1: Liver Kinase B1, AMPK: AMP-Activated Protein Kinase. Created in BioRender.

Nearly 25 years of literature consistently demonstrates that there is no single unifying mechanism of action of metformin in cancer. As a normoglycemic agent for type 2 DM, metformin decreases hepatic gluconeogenesis and lipid synthesis, decreases adipose tissue fatty acid synthesis and lipolysis, decreases pancreatic insulin secretion, and increases muscle glucose uptake [111,112] (Figure 1). This can occur either through liver kinase B1 (LKB1)/AMPK activation in target tissues or a direct inhibition of insulin signaling.

**Figure 2:**
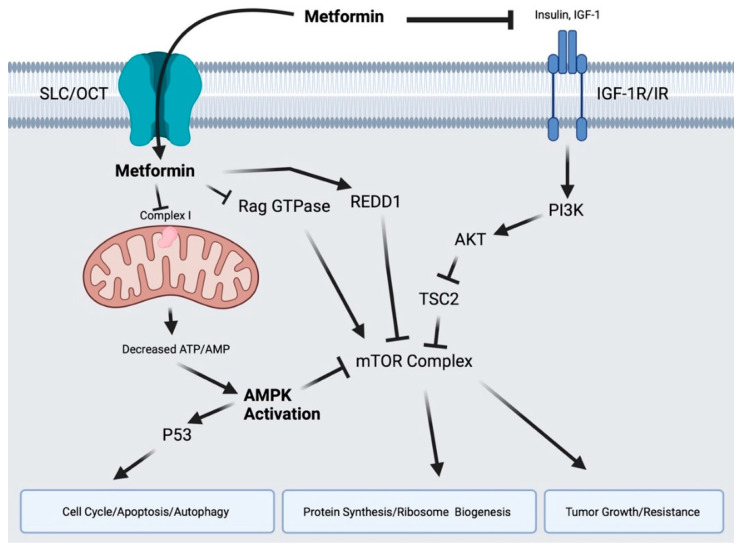
Molecular effects of metformin in cancer cells. Metformin directly inhibits complex I of the electron transport chain in the mitochondria resulting in decreased ATP/AMP ratio and activation of AMPK. AMPK activation inhibits mTOR and activates P53 to impact subsequent cellular processes. Metformin also inhibits mTOR in an AMPK-independent manner, through Rag GTPases and REDD1. Reduced insulin availability through metformin’s systemic effects indirectly modulates the proliferative pathway, PI3K/AKT. AMP: Adenosine Monophosphate; AMPK: AMP-Activated Protein Kinase; ATP: Adenosine Triphosphate; IGF: Insulin-like Growth Factors; IGF-R: Insulin-like Growth Factor Receptor; mTOR: Mammalian Target of Rapamycin; OTC: Organic Cation Transporter; PI3K: Phosphoinositide 3-kinase; REDD1: Regulated in Development and DNA damage responses 1; SLC: Solute Carrier Transporter; TSC2: Tuberous Sclerosis Complex 2. Created in BioRender.

Next, we will briefly summarize the well-established activity of metformin in cancer that has been recently reviewed [48,86,87] and as summarized in Figure 2. We will then shift to novel mechanisms of action established over the last five years, including immunomodulatory and epigenetic effects of metformin.

Metformin’s well-established anti-cancer mechanisms involve direct and indirect, AMPK-dependent and -independent inhibition of mammalian target of rapamycin (mTOR), which plays a significant role in promoting tumor proliferation as well as inhibiting apoptosis and autophagy. The indirect, AMPK-independent inhibition of mTOR stems from metformin’s ability to decrease systemic insulin [113,114,115,116]. Decreased insulin leads to decreased signaling through the phosphoinositide 3-kinase (PI3K)-protein kinase B (AKT) pathway, subsequently allowing tuberous sclerosis complex 2 (TSC2) to inhibit mTOR [117]. Metformin is also taken up by cancer cells through organic cation transporters [118] and subsequently inhibits complex I of the mitochondrial electron transport chain leading to decreased oxidative phosphorylation [119]. The decreased ratio of adenosine triphosphate (ATP) to adenosine monophosphate (AMP) leads to cellular stress, activation of AMPK [120,121,122,123], and downstream inhibition of mTOR kinase activity, which results in a decrease in protein synthesis, cell growth, and proliferation [117,124,125,126,127]. Early on, metformin’s role in cancer clearly showed that AMPK-dependent inhibition of mTOR is required for multiple anti-cancer effects, as the phenotype can be rescued by targeting AMPK with siRNA or Compound C as well as constitutive activation of mTOR and short hairpin RNA targeting TSC2 [117,124,125,128,129]. Furthermore, multiple reports have demonstrated that metformin can also activate AMPK indirectly through activation of upstream energy sensor, LKB1, or via ataxia telangiectasia mutated (ATM) [130,131,132]. Metformin also inhibits mTOR independently of AMPK through activation of DNA-damage-inducible transcript 4 (REDD1), which inhibits mTOR via TSC2 activation [133], or via inhibition of Rag GTPases [134]. Metformin’s inhibition of Rag GTPases was independent of amino acid levels, which have previously been shown to control Rag GTPases and downstream mTOR activity [135,136].

Metformin’s mechanism of action also involves regulation of additional transcription factors, such forkhead box O3a (FOXO3a), mitogen-activated protein kinase (MAPK), Sonic hedgehog, Wnt, Notch, and Kruppel-like factor 5 [103,110,137,138]. FOXO3a upregulation by metformin is particularly interesting given FOXO3a’s ability to induce MAPK-dependent expression of the mitochondrial genome to support mitochondrial metabolism. In fact, activation of FOXO3a has been shown to be necessary for metformin’s pro-apoptotic and chemosensitizing effects in multiple tumor models by allowing metformin to promote mitochondrial biogenesis while simultaneously inhibiting complex I activity [137,138]. It is these multifaceted aspects of metformin that make it a unique drug and encourages further elucidation of its anti-cancer mechanism of action to identify optimal drug combinations to effectively target cancer cells.

### 3.3. Immunomodulatory Effects of Metformin

More recently, metformin has been found to exhibit antitumor activity through regulation of the immune response to cancer. Multiple studies have found metformin can decrease programmed death-ligand 1 (PD-L1) on tumor cells through both AMPK-dependent [139,140,141] and AMPK-independent [142,143] mechanisms, resulting in enhanced cytotoxic T lymphocyte activity. However, this anti-PD-L1 activity may be tissue-dependent. In a NSCLC model, the inverse was found to be true in which LKB1-overexpression actually increased PD-L1 in an AMPK-dependent fashion [144]. As a result, LKB1-intact NSCLC tumors could be sensitized to anti-PD-1 antibodies with metformin whereas no obvious suppression from metformin was observed in LKB1-deficient tumors [144].

Metformin may also act directly on cytotoxic T cells to augment their anti-cancer activity. Metformin administration induces interferon-gamma (IFN-γ) production in CD8+ tumor infiltrating lymphocytes in multiple solid tumor models [145,146,147]. Furthermore, metformin inhibited accumulation and suppressive activity of myeloid-derived suppressor cells, which are a major immunosuppressive cell type that inhibits T-cells and promotes tumor immune escape [145,148]. Interestingly, metformin is detrimental to CD19-chimeric antigen receptor-modified T cells as it inhibits proliferation and cytotoxicity while inducing apoptosis via AMPK activation and downstream suppression of mTOR [149]. Thus, the T-cell targeting properties of metformin may be context- and cancer subtype-dependent. 

In addition to T cell regulation, metformin can enhance natural killer (NK) cell cytotoxicity of human cervical cancer cells by altering tumor cell surface expression of NK-cell ligands via the PI3K/AKT pathway, leading to increased NK cell activation [106]. Furthermore, direct exposure of NK cells to metformin enhances their cytolytic activity and increases NK cell tumor infiltration independently of AMPK [150]. Metformin also directly and indirectly modulates macrophage-targeting of tumor cells. Metformin represses CD47 gene expression in a miRNA-708-dependent manner to allow macrophage phagocytosis of breast cancer stem cells [105]. Furthermore, metformin modulates expression of macrophage-related cytokines, thereby suppressing the ability of cancer cells to promote the protective macrophage 2 phenotype and promoting the anti-cancer macrophage 1 phenotype in an AMPK/NF-κB-dependent manner [151,152].

### 3.4. Epigenetic Regulation of Metformin

Epigenetic mechanisms, such as hypermethylation of tumor suppressor genes, general hypomethylation of the genome, and alterations in histone posttranslational modifications, play a role in tumorigenesis and therapy resistance [153]. Recent studies indicate that metformin can target cancer cells through epigenetic modifications. Metformin-activated AMPK has been demonstrated to increase global DNA methylation in colon, breast, and endometrial cancer cells [154,155,156]. Altered DNA methyltransferase (DNMT) activity by metformin also contributed to anti-cancer activity by regulating long non-coding RNAs [157,158]. In two studies, metformin has been found to regulate epigenetics specifically through targeting the oncometabolite 2-hydroxyglutarate (2HG) [159,160]. Interestingly, in one study this was through the traditional route of targeting IDH1/2 mutations in endometrial cancer [160]. In another study, the 2HG oncometabolite was found to be elevated in breast cancer in vitro and in vivo in the absence of IDH1/2 mutations [159]. Metformin specifically inhibited 2HG production in this model through knockdown of phosphoglycerate dehydrogenase in an AMPK-dependent manner leading to anti-cancer activity [159]. Additional work has demonstrated that metformin can also suppress epigenetic modifier, enhancer of zeste homolog 2 (EZH2), in its anti-cancer activity in prostate adenocarcinoma and neuroendocrine tumors [161,162]. Metformin can also target histone acetylation to antagonize melanoma progression [163]. 

## 4. Conclusive Remarks

Preclinical studies have consistently demonstrated antineoplastic effects of metformin. Additionally, observational and epidemiological studies have reported lower incidence and mortality rates of cancer in patients taking metformin. However, these results have translated to modest benefits in clinical trials, which may be attributed to several hypotheses that can guide future research. The inherent limitations of observational and retrospective study designs can be a source of potential bias leading to an overestimation of the benefits of metformin in patients. Moreover, while preclinical models have been key in characterizing the antineoplastic mechanisms of metformin, they suffer from several limitations that impact their translation to the clinic. Some authors have argued that metformin concentrations used in preclinical studies were significantly higher than the plasma concentrations reached in clinical trials [10]. Additionally, in vivo models require optimization to recapitulate tumor heterogeneity, including cancer stem cells [164], and the immuno- and micro-environments to better predict clinical results [165]. 

Of note, many of the relevant clinical trials either recruited a small number of patients or enrolled patients with an advanced cancer stage, both of which can confound results. To optimize the design of clinical trials, additional research is required to identify key factors (both patient- and tumor-related) that affect metformin sensitivity. For example, the insulin-lowering effect of metformin is thought to contribute to its anti-cancer activity, which suggests that patients with hyperinsulinemia or tumors expressing the insulin receptor, LKB1, or TSC2 may benefit most from metformin [166].

## Figures and Tables

**Table 1:** Metformin’s biometric information.

Characteristics	Metformin
Structural name	3-(diaminomethylidene)-1,1-dimethylguanidine
Structure	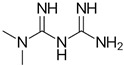
Formula	C4-H11-N5
Molecular weight	129.16 g/mol
Density	1.3 g/cm^3^
Melting point	223–226 °C
Boiling point	224.1 °C at 760 mmHg
Color	White
CAS number	657-24-9
PubChem Substance ID	4091

**Table 2:** Recent clinical trials investigating oral metformin use in cancers [11,12,13,14,15,16,17,18,19,20,21,22,23,24,25,26,27,28,29,30,31,32,33,34,35,36,37,38,39,40,41,42,43,44,45,46,47,48]. N/A: not applicable.

Tumor Location	Trial Reference	ID/ Phase	Tumor/Patient Characteristics	Number of Participants	Treatment	Result	Other Comments
Various Solid Tumors	[38]	Phase Ib	Variety of advanced solid tumors refractory to standard therapies	9	Everolimus + metformin (*n* = 9; metformin 500 mg twice daily)	Combination therapy was poorly tolerated	Open-label, prospective, single-center, dose-escalation study, The Netherlands
	[39]	--	Variety of advanced solid tumors (metastatic or unresectable)	24	Sirolimus + metformin (*n* = 11; maintenance on 1000 mg once daily) vs. sirolimus (*n* = 13)	Combination therapy did not improve mTOR inhibition	Open-label, randomized
	[40]	NCT01442870 Phase I	Variety of solid tumors (nondiabetic, histologically confirmed solid tumors receiving adjuvant or systemic chemotherapy)	100	Concurrent chemotherapy + metformin (*n* = 49; 500 mg twice daily) vs. delayed chemotherapy + metformin (*n* = 51; 500 mg twice daily)	Metformin is safe to use in combination with a wide range of chemotherapy regimens	Delayed-start, randomized
	[41]	NCT02496741 Phase Ib	*IDH1*-mutated solid tumors including chondrosarcoma (refractory grade II-III), glioma (WHO grade II-IV), and intrahepatic cholangiocarcinoma	17	Chloroquine + metformin (*n* = 17; maximum of 1500 mg twice daily)	Combination treatment with chloroquine and metformin did not induce clinical response	Prospective, open-label, dose-escalation, The Netherlands
Glioma	N/A	NCT04945148 Phase II	Glioblastoma, IDH-wildtype	640	Metformin (1500–3000 mg daily) plus radiation and temozolomide	No results available	Open-label, prospective, single-center, France
	N/A	NCT02149459 Phase I	Brain neoplasms	18	Metformin (dose not specified), radiation, and low carbohydrate diet	No results available	Open-label, prospective, single-center, Israel
	N/A	NCT02780024 Phase II	Glioblastoma	50	Metformin (dose not specified) and neoadjuvant temozolomide followed by combined radiation and temozolomide	No results available	Open-label, prospective, single-center, Canada
	N/A	NCT03243851 Phase II	Recurrent or refractory glioblastoma	81	Metformin (ramp up to 2000 mg daily) and low dose temozolomide	No results available	Open-label, prospective, single-center, South Korea
	N/A	NCT03151772 Phase I	Glioblastoma	3	Metformin (850 mg daily) and disulfiram for 3 days preoperatively	No results available, study was terminated for low enrollment	Open-label, prospective, single-center, Sweden
	N/A	NCT04691960 Phase II	Glioblastoma	36	Metformin (ramp up to 850 mg three times daily) and ketogenic diet	No results available	Open-label, prospective, single-center, US
	N/A	NCT05183204 Phase II	Glioblastoma	33	Metformin (ramp up to 850 mg three times daily as tolerated), ketogenic diet and Paxalisib|	No results available	Open-label, prospective, single-center, US
	N/A	NCT01430351 Phase I	Glioblastoma and gliosarcoma	144	Metformin (dose not specified), mefloquine, memantine, hydrochloride, hydrochloride, and temozolomide	No results available	Open-label, prospective, single-center, US
Bladder Tumors	[26]	NCT03379909 Phase II	Non-muscle-invasive bladder cancer (intermediate-risk)	49 (target)	Metformin (maximum of 3000 mg daily)	Ongoing	Multicenter, open-label
Breast Tumors	[16]	NCT00490139 Phase III	HER2-positive primary breast cancer	8381	Substudy analysis of diabetic study participants on/off metformin therapy (dose not specified; all patients previously taking for DM) in patients receiving relevant anti-HER2 therapies, described elsewhere	Diabetic patients with HER2-positive breast cancer demonstrated better outcomes when treated with metformin compared to diabetic breast cancer patients not on metformin, whereas outcomes of patients with HR-negative status were not affected by diabetes treatment status	Randomized, adjuvant trial
	[11]	NCT01654185 Phase II	Hormone receptor positive locally advanced or metastatic breast cancer	60	Aromatase inhibitor (exemestane or letrozole) + metformin (*n* = 30; maintenance on 500 mg daily) vs. aromatase inhibitor (exemestane or letrozole) + placebo (*n* = 30)	No improved efficacy was observed in the addition of metformin to aromatase inhibitor treatment	Randomized, China
	[42]	NCT01266486 Phase I	Treatment-naïve primary breast cancer	40	Metformin (*n* = 40; maintenance on 1500 mg daily)	Metformin treatment precipitated two distinct metabolic responses in tumors	Window study design, UK
	[14]	NCT01310231 Phase II	Metastatic breast cancer (nondiabetic)	40	Chemotherapy + metformin (*n* = 22; maintenance on 850 mg daily) vs. chemotherapy + placebo (*n* = 18)	Combined chemotherapy with metformin had no demonstrated effect on PFS, OS, or RR	Randomized, double-blind, Canada
	[12]	NCT01885013 Phase II	Metastatic breast cancer (HER2-negative, nondiabetic)	122	Chemotherapy (doxorubicin + cyclophosphamide) + metformin (*n* = 57; maintenance on 2000 mg daily) vs. chemotherapy (doxorubicin + cyclophosphamide) (*n* = 65)	The addition of metformin did not provide a meaningful clinical benefit to PFS or OS but was found to decrease the incidence of severe neutropenia	Open-label, multicenter, randomized
	[13]	NCT01650506 Phase I	Metastatic triple negative breast cancer who had received at least one prior therapy	8	Erlotinib + metformin (*n* = 8; maximum dose was 850 mg thrice daily)	Combination therapy was well-tolerated but did not result in objective tumor response	USA
	[15]	IRCT20100706004329N7	Breast fibroadenoma (nondiabetic)	175	Metformin (*n* = 83; maximum dose was 1000 mg daily) vs. placebo (*n* = 92)	The effect of metformin is most obvious in smaller masses and appears to have a favorable effect compared to placebo in terms of reducing chances of significant enlargement of tumors	Iran
	[17]	NCT01627067 Phase II	Metastatic, hormone receptor-positive, HER2-negative breast cancer (obese or overweight, postmenopausal)	22	Everolimus + exemestane + metformin (*n* = 22; 1000 mg twice daily)	This treatment combination had moderate clinical benefit	USA
Colorectal Tumors	[24]	--	Stage II-III colon cancer	120 out of total 3759 enrolled in TOSCA	Goal of original TOSCA study was to compare 3- vs. 6-month treatment with fluoropyrimidine-oxaliplatin adjuvant chemotherapy (post-resection) ■ Metformin users (*n* = 76; dose not specified) ■ Metformin nonusers (*n* = 44)	Neither metformin use, nor DM, nor metformin dosage were associated with OR/RFS	Subanalysis
	[23]	NCT01312467 Phase IIa	Nondiabetic, obese patients with recent history of colorectal adenoma	32	Metformin (*n* = 32; maintenance on 1000 mg twice daily)	Metformin intervention did not reduce rectal mucosa pS6 (marker of polyp suppression) or Ki-67 (marker of proliferation) levels	USA
	[25]	Phase II	Refractory colon cancer	41	Irinotecan + metformin (*n* = 41; maintenance on 2500 mg daily)	Irinotecan/metformin was able to provide disease control, with diarrhea as a significant side effect	Single-center
Lung Tumors	[18]	NCT01864681 Phase II	Non-small cell lung cancer (locally advanced, stage IIIb-IV, EGFR mutated, treatment-naïve, nondiabetic)	224	Gefitinib + metformin (*n* = 100; maintenance on 1000 mg twice daily) vs. gefitinib + placebo (*n* = 100)	Combination treatment resulted in non-significantly worse outcomes and was accompanied by more side effects (diarrhea)	Multicenter, double-blind, China
	[22]	NCT01578551 Phase II	Chemo-naïve or metastatic nonsquamous NSCLC (stage IIIB or IV; nondiabetic)	25	Carboplatin + paclitaxel + bevacizumab + metformin (*n* = 19; 1000 mg twice daily) vs. carboplatin + paclitaxel + bevacizumab (*n* = 6)	The metformin combination treatment group experienced increased PF	Single center, open-label, USA
	[19]	NCT03071705 Phase II	Lung adenocarcinoma (EGFR-mutated, stage IIIb-IV)	139	EGFR-TKI (erlotinib, afatinib, or gefitinib) + metformin (*n* = 69; 500 mg twice daily) vs. EGFR-TKI (erlotinib, afatinib, or gefitinib) (*n* = 70)	The addition of metformin to EGFR-TKI standard therapy significantly improved PFS and OS in advanced lung adenocarcinoma patients	Randomized, open-label, prospective, Mexico
	[20]	NCT02186847 Phase II	NSCLC (unresectable, stage III; nondiabetic)	167	Chemoradiation + metformin (*n* = 86; maintained on 2000 mg daily) vs. chemoradiation (*n* = 81)	There was no survival benefit associated with metformin addition to traditional chemoradiation therapy	Randomized, open-label, multicenter, international
	[21]	NCT02115464 Phase II	Locally advanced NSCLC (nondiabetic)	54	Chemoradiation (platinum-based) + metformin (*n* = 26; maintained on 2000 mg daily) vs. chemoradiation (platinum-based) (*n* = 28)	Trial was stopped early due to low accrual; the addition of metformin to chemoradiotherapy was associated with a worse treatment outcome and increased toxicity	Randomized, open-label, multicenter, Canada
Ovarian Tumors	[27]	ChiCTR-IOR-17011859	Epithelial ovarian cancer (nondiabetic)	47	Debulking + paclitaxel/carboplatin + metformin (*n* = 20; 850 mg daily) Debulking + paclitaxel/carboplatin (*n* = 24)	There was no evidence of metformin effect on PFS	China
	[29]	NCT02312661 Phase I	Advanced epithelial ovarian cancer (FIGO III-IV)	15	Paclitaxel/carboplatin + metformin (*n* = 15; maximum dose of 1000 mg thrice daily)	The recommended phase II dose is 1000 mg thrice daily and there is a potential pharmacokinetic interaction between metformin and carboplatin, though the combination is well-tolerated	Dose escalation study, the Netherlands
	[28]	NCT01579812 Phase II	Advanced-stage (IIC/III/IV) epithelial ovarian cancer (nondiabetic)	38 evaluable	Neoadjuvant metformin + debulking surgery + adjuvant chemotherapy plus metformin (*n* = 23; maintenance on 1000 mg twice daily) vs. neoadjuvant chemotherapy and metformin + interval debulking surgery + adjuvant chemotherapy plus metformin (*n* = 15)	Addition of metformin is associated with better OS and a significant cancer stem cell population reduction	USA
Prostate Tumors	[43]	EudraCT number 2014–005193-11	Prostate cancer (newly diagnosed, localized, scheduled for radical prostatectomy)	100	Metformin (*n* = 50; maintenance on 1000 mg twice daily) vs. placebo (*n* = 50)	Ongoing	Randomized, placebo-controlled, double-blind, window of opportunity, UK
	[30]	NCT01677897 Phase II	Prostate cancer (metastatic, castration-resistant, with PSA progression while on abiraterone therapy)	25	Abiraterone + metformin (*n* = 25; 1000 mg twice daily)	Combination therapy resulted in no clinical benefit and did not affect progression; higher-than-expected gastrointestinal toxicity was also reported	Pilot study, Switzerland
	[31]	NCT01796028 Phase II	Prostate cancer (metastatic, castration-resistant, nondiabetic)	99	Docetaxel + metformin (*n* = 50; 850 mg twice daily) vs. docetaxel + placebo (*n* = 49)	No improvement was observed in metformin group vs. placebo	French, prospective, multicenter, randomized, placebo-controlled
	[32]	NCT02614859 Phase II	Prostate cancer (nondiabetic, recurrent PC, overweight or obese with BMI > 25)	29	Bicalutamide + metformin (*n* = 20; 1000 mg twice daily) vs. bicalutamide (*n* = 9)	This study was ended early due to predicted inability to reach its primary endpoint (achievement of undetectable PSA at 32 weeks)	Randomized, open-label, USA
Skin Tumors	[44]	NCT02325401	HNSCC	39	Metformin (*n* = 39; maintenance on 2000 mg daily)	Metformin is capable of modulating the HNSCC microenvironment	Window of opportunity (post-biopsy, pre-resection)
	[33]	NCT01840007 Phase I	Metastatic melanoma (patients who progressed after first-line treatment and were not eligible or did not respond to ipilimumab)	17	Metformin (*n* = 17; 1000 mg thrice daily)	Metformin shows no efficacy and poor safety in treating metastatic melanoma	Multicenter, pilot, prospective, open-label, France
	[45]	NCT02083692	HNSCC (nondiabetics)	50	Metformin (*n* = 49; maintenance on 1000 mg twice daily)	Metformin treatment alters the immune tumor microenvironment, regardless of HPV status	Non-randomized
	[46]	NCT02325401 Phase I	Locally advanced HNSCC (nondiabetic, stage III-IV)	20	Cisplatin + radiotherapy + metformin (*n* = 20; maximum dose was 3000 mg daily)	Cisplatin did not appear to affect metformin pharmacokinetics	USA
	[47]	NCT02581137 Phase IIa	Oral premalignant lesions (nondiabetic)	26	Metformin (*n* = 26; maintenance on 2000 mg daily)	Metformin treatment was associated with good histological response and decreased mTOR activity	Open-label
	[48]	NCT02083692	HNSCC	50	Metformin (*n* = 39 completed; maintenance on 1000 mg twice daily)	Metformin treatment alters the immune tumor microenvironment and results in increased apoptosis in HPV-, tobacco+ HNSCC patients compared to HPV+ HNSCC patients	USA
Uterine Tumors	[34]	Phase III	Endometrioid endometrial cancer or atypical endometrial hyperplasia (pre-surgery)	88	Metformin (*n* = 45; maintenance on 850 mg twice daily) vs. placebo (*n* = 43)	Pre-surgical treatment with metformin does not reduce tumor proliferation	Multicenter, randomized, double-blind, pre-surgical window study design, UK
	[36]	NCTO1877564	Endometrial cancer (nondiabetic, obese, pre-surgery)	13	Metformin (maintenance at 850 mg twice daily)	Pre-surgical treatment with metformin alters steroid receptor signaling of EC cells	Window design
	[37]	jRCT2031190065	Endometrial cancer	120 (target)	Medroxyprogesterone acetate vs. medroxyprogesterone acetate + metformin (750 mg daily) vs. medroxyprogesterone acetate + metformin (1500 mg daily)	Ongoing	Prospective, randomized, open, blinded-endpoint, dose–response, multicenter, Japan
	[35]	NCT03618472	Endometrial cancer (nondiabetic)	49	Metformin (*n* = 25; 850 mg daily) vs. placebo (*n* = 24)	Pre-surgical metformin treatment significantly decreased proliferative tissue marker Ki-67	Randomized, double-blind, placebo-controlled, Thailand
Leukemia	N/A	NCT01324180 Phase I	Relapsed acute lymphoblastic leukemia	14	Metformin (twice daily in dose escalation schema) in combination with vincristine, dexamethasone, PEG-asparaginase, doxorubicin, and intrathecal cytarabine	Completed	Single group assignment, interventional, dose-escalating, open-label
	N/A	NCT01849276 Phase I	Relapsed/refractory acute myeloid leukemia	2	Metformin (twice daily in dose escalation schema on days 1–15) + intravenous cytarabine	Terminated (due to slow accrual)	Single group assignment, interventional, open-label
Lymphoma	N/A	NCT03200015 Phase II	Diffuse large B-cell lymphoma (DLBCL)	15	Metformin (ramp up to 850 mg thrice daily) + rituximab, cyclophosphamide, doxorubicin, vincristine, prednisone	Unknown	Single group assignment, interventional, open-label
	N/A	NCT02531308 Phase II	DLBCL	5	Metformin (ramp up to 850 mg twice daily) + rituximab, cyclophosphamide, doxorubicin, vincristine, prednisone, pegfilgrastim	Terminated (slow accrual)	Single group assignment, interventional, open-label
Myeloma	N/A	NCT03829020 Phase I	Recurrent plasma cell myeloma and refractory plasma cell myeloma	36	Metformin (dose escalation schema) + bortezomib, nelfinavir	Recruiting	Single group assignment, interventional
	N/A	NCT02948283 Phase I	Recurrent plasma cell myeloma and refractory plasma cell myeloma	3	Metformin (twice daily in dose escalation schema) + ritonavir	Completed	Single group assignment, interventional

## Data Availability

Not applicable.
